# Supplementary material for: In Vitro Activity of the Arylaminoartemisinin GC012 against Helicobacter pylori and Its Effects on Biofilm
Source: Pathogens. 2022 Jun 29;11(7):740. doi: 10.3390/pathogens11070740 (PMC9324866; doi:10.3390/pathogens11070740)
Supplement: Supplementary file 1 [file pathogens-11-00740-s001.zip › pathogens-1761300-supplementary.pdf]

Supplementary Material

In Vitro Activity of the Arylaminoartemisinin GC012 against *Helicobacter pylori* and Its Effects on Biofilm

Francesca Sisto [1,*], Simone Carradori [2], Sarah D'Alessandro [3], Nadia Santo [4], Norma Lattuada [4], Richard K. Haynes [5], Donatella Taramelli [3] and Rossella Grande [2]

1 Department of Biomedical, Surgical and Dental Sciences, University of Milan, Via Pascal 36, 20133 Milan, Italy
2 Department of Pharmacy, "G. d'Annunzio" University of Chieti-Pescara, Via dei Vestini 31, 66100 Chieti, Italy; simone.carradori@unich.it (S.C.); rossella.grande@unich.it (R.G.)
3 Department of Pharmacological and Biomolecular Sciences, University of Milan, Via Pascal 36, Milan, 20133, Italy; sarah.dalessandro@unimi.it (S.D.); donatella.taramelli@unimi.it (D.T.)
4 UNITECH- University Technological Platform, University of Milan, Via Golgi 19, 20133 Milan, Italy; nadia.santo@unimi.it (N.S.); norma.lattuada@unimi.it (N.L.)
5 Centre of Excellence for Pharmaceutical Sciences, Faculty of Health Sciences, North-West University, Potchefstroom 2520, South Africa; haynes@ust.hk
* Correspondence: francesca.sisto@unimi.it

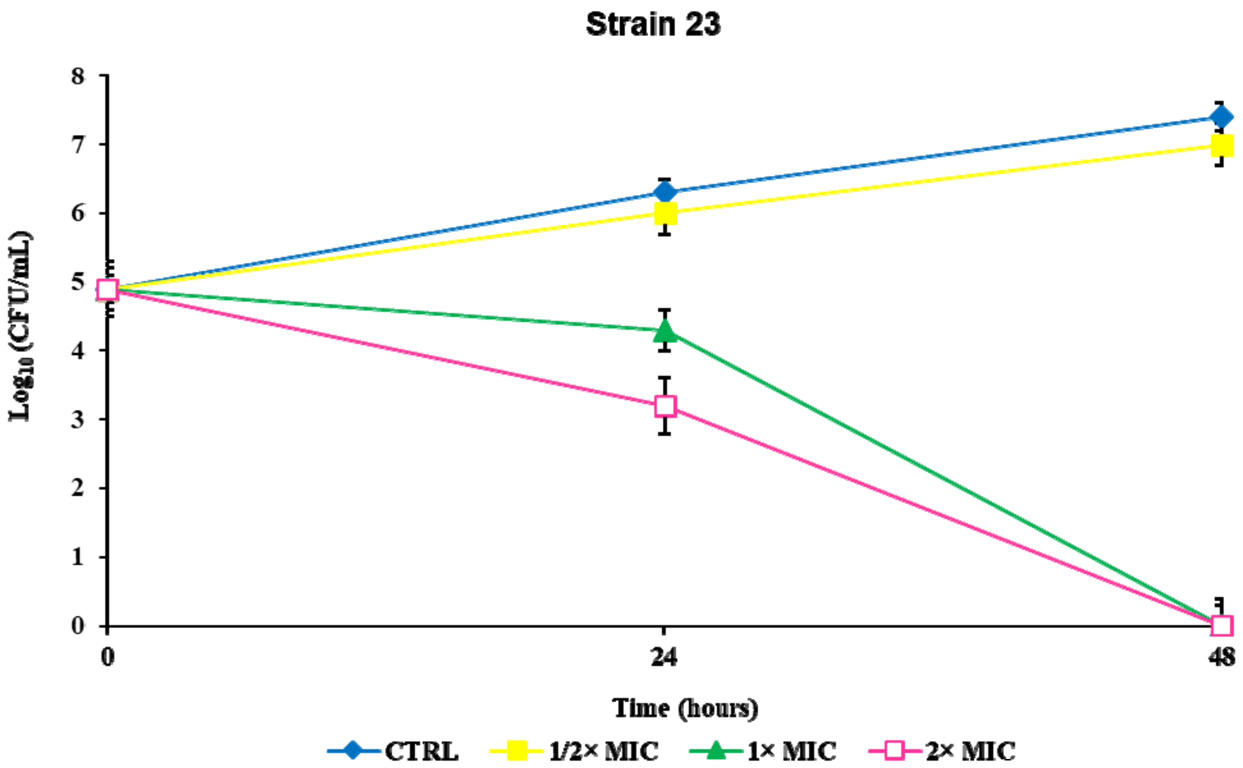

Figure S1. Kinetic of the killing activity of *H. pylori* clinical isolate strain 23 by DHA. Antibacterial activity was evaluated in RPMI Megacell with 3% FCS in the presence or absence of the indicated drugs concentrations for different length of time. The data are expressed as mean CFU±SD recovered from three different experiments in triplicate.

A

B

C

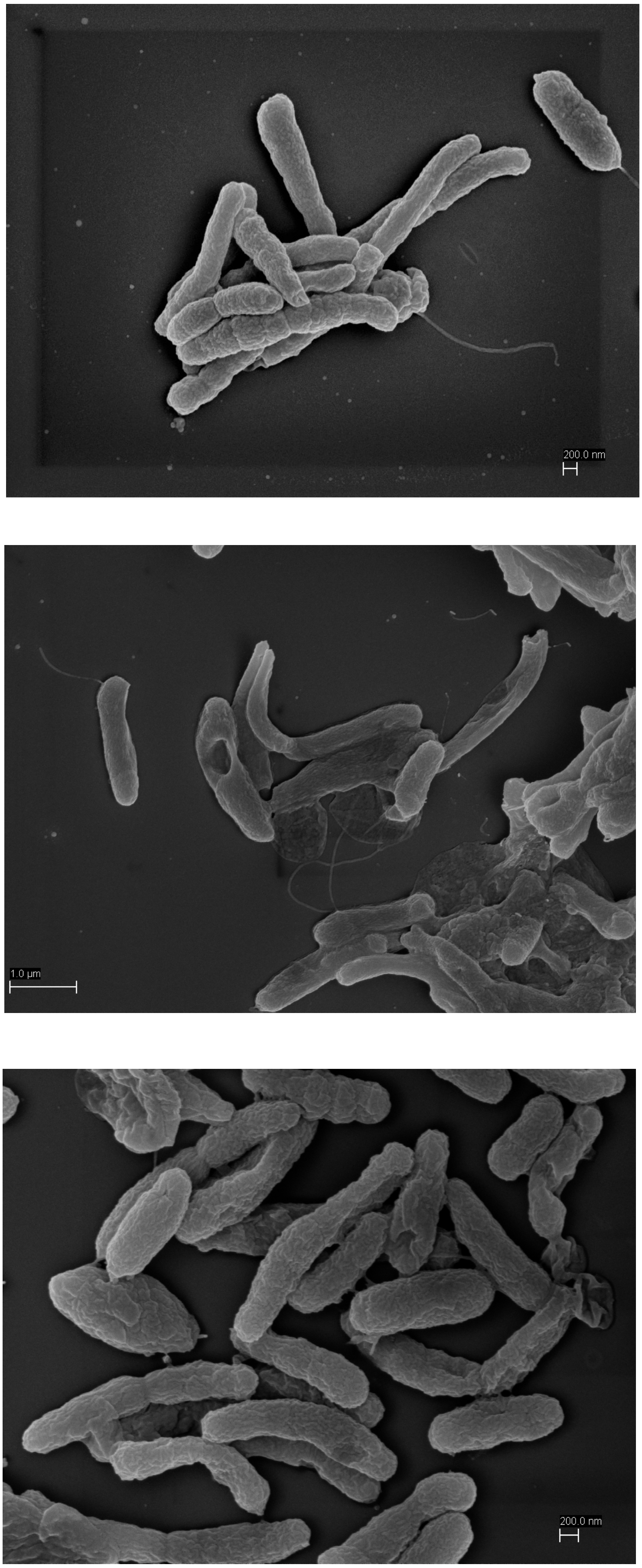

Figure S2. SEM analysis of *H. pylori* after treatment with GC012 and DHA. *H. pylori* were sampled after 24 h of exposure to medium (**A**); to MIC concentration of GC012 (**B**); to MIC concentration of DHA (**C**).

A

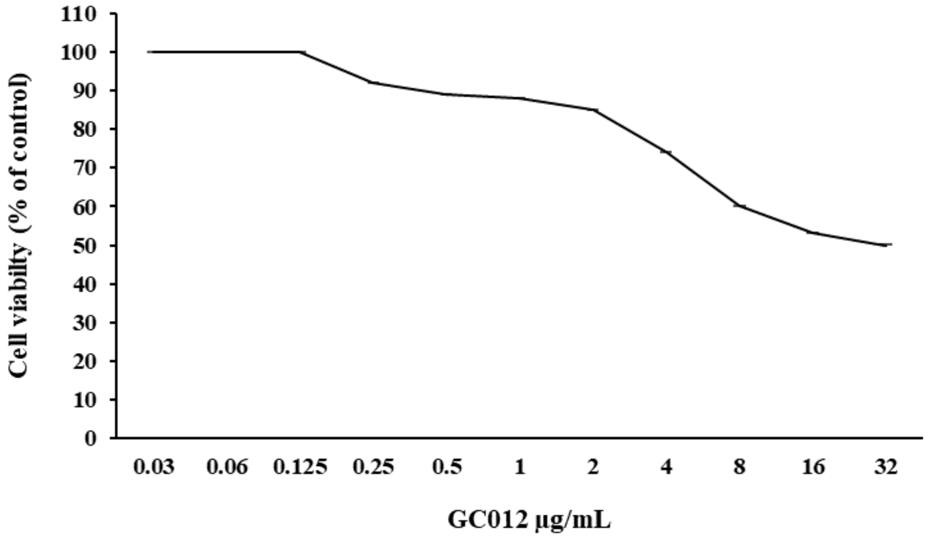

B

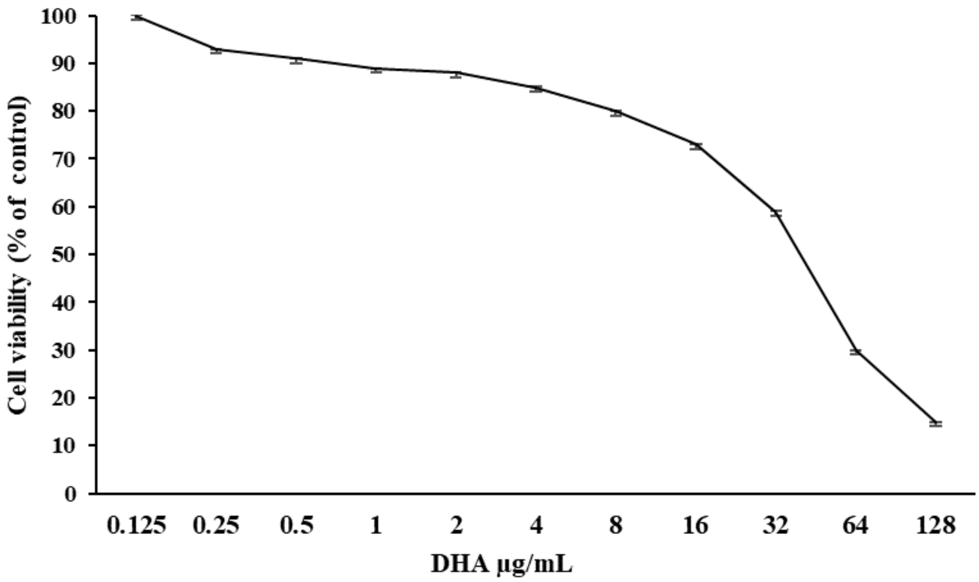

Figure S3. Cell viability after treatment with GC012 and DHA by MTT assay. GES-1 cells were incubated for 24 h with different doses of GC012 (**A**) and DHA (**B)** in RPMI medium as described in Material and Methods section. The effect is expressed as percentage of control (untreated culture) of the optical density (OD) ± SD.

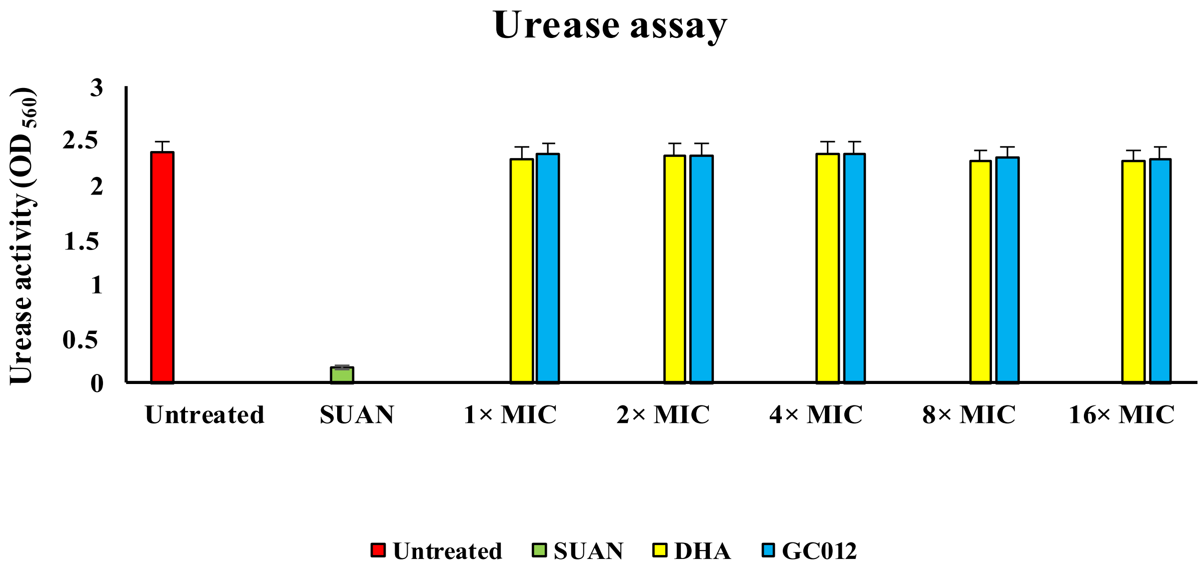

Figure S4. Effect of DHA and GC012 on urease production. The urease activity was measured on 96 well plates, after incubation of clinical strain 23 with different concentration of DHA (MIC 0.5 μg/mL) and GC012 (MIC 0.06 μg/mL). Untreated samples (0 μg/mL), and urease inhibitor SUAN (32 μg/mL) [52] treated samples were used as positive and negative control, respectively. The ammonia concentration was recorded by spectrophotometer at 560 nm.
